# Identification of mitogen-activated protein kinase phosphatase-1 (MKP-1) protein partners using tandem affinity purification and mass spectrometry

**DOI:** 10.1007/s43440-023-00471-7

**Published:** 2023-03-24

**Authors:** Ewelina Fic, Agata Cieślik, Małgorzata Figiel, Marta Dziedzicka-Wasylewska

**Affiliations:** Department of Physical Biochemistry, Faculty of Biochemistry, Biophysics and Biotechnology, Jagiellonian University, Gronostajowa 7, 30-387 Cracow, Poland

**Keywords:** Depression, Affinity chromatography, Tags, Interactomics, PC12 cell line

## Abstract

**Background:**

According to the World Health Organization Report, depressive disorders affect about 10% of the population. The molecular mechanism of the pathogenesis of depression is still not well understood. The new findings point to phosphatases as potential targets for effective depression therapy. The aim of the present work was the development of a method that would enable the identification of mitogen-activated protein kinase phosphatase-1 (MKP-1) protein partners using a proteomic approach.

**Methods:**

The research was carried out using the PC12 cell line, often used as a model for neurobiological research. The use of the procedure for efficient purification of protein complexes—tandem affinity purification (TAP) will facilitate the identification of proteins interacting with MKP-1, a potential goal of effective antidepressant therapy.

**Results:**

Identified proteins belong to various groups: cytoskeletal, ribosomal, nucleic acid binding, chaperones, and enzymes and may potentially be involved in the molecular mechanism of depression.

**Conclusions:**

The presented protocol for the purification of protein complexes is universal and can be successfully used in different mammalian cell lines. Proteins identified in the present work have been reported in the literature concerning studies on depressive disorders, which speaks in favour of their role in depression.

**Supplementary Information:**

The online version contains supplementary material available at 10.1007/s43440-023-00471-7.

## Introduction

Depression is a severe mental disorder characterized by low mood as well as cognitive and executive dysfunction. The molecular mechanisms underlying this illness or the effects of antidepressants are still unknown. It seems that advanced biochemical studies may contribute to a better understanding of the etiopathogenesis of depression. It is estimated that about 80% of proteins perform their functions in complexes with other proteins. These protein–protein interactions (protein–protein interactions—PPIs) are examined by interactomics. The purification strategy of protein complexes, defined as Tandem Affinity Purification, TAP, in combination with mass spectrometry, allows the purification of native protein complexes and the identification of interacting partners [1, 2]. TAP is a strategy that allows the isolation of protein complexes from various tissues. This method is based on the use of 2–3 different tags attached to the tested protein and the introduction of the construct into host cells [3]. Thanks to the combination of tags from the protein mixture, “native” complexes of a given protein with other proteins, using the affinity chromatography method, are extracted [4, 5].

The literature reports have suggested the important role of Mitogen-Activated Protein Kinase Phosphatase-1 (MKP-1) in the pathogenesis of depression and other neuropsychiatric disorders [6, 7]. MKP-1 belongs to a family of proteins that dephosphorylate serine/threonine and tyrosine amino acid residues [8]. The MKP-1 protein was identified as a negative regulator of the MAPK (Mitogen-Activated Protein Kinase) cascade [6], which is associated with the occurrence of depressive symptoms. Experiments carried out using the animal model of depression show that the increase in mkp-1 gene expression in the hippocampus leads to the occurrence of depressive symptoms [9]. Chronic treatment with antidepressants normalizes symptoms caused by excess MKP-1, and mice lacking the mkp-1 gene are resistant to stress [6]. Post-mortem studies on the brains of people diagnosed with depression also showed an elevated level of MKP-1 expression and associated with ERK (Extracellular Signal-Regulated Kinases) signal inhibition. Administration of sanguinarine, a selective MKP-1 inhibitor [10, 11] to the ventrolateral orbital cortex in rats significantly reduces depressive behaviour in a dose-dependent manner compared to the control group. Decreased MKP-1 expression and increased ERK activation were also observed. MKP-1 inhibition may have antidepressant effects, as confirmed by behavioural tests with effects similar to those observed after fluoxetine treatment [12]. The results indicate that within the orbital cortex, MKP-1 is involved in the development of depression and may be a potential target for the pharmacotherapy of depression [11].

Recently published results indicate that other factors (not yet identified) may be critical for elevated MKP-1 levels and thus result in depressive symptoms [7, 13].

The purpose of the presented study was the optimization of the TAP strategy aimed at identifying MKP-1 protein partners. In the study the production of MKP-1 with Strep II FLAG -tag (SF-tag) was carried out on the cell line PC12 (rat adrenal pheochromocytoma cell line)—this line is a useful model for neurobiological and neurochemical research [14] and is successfully used in interactomic studies [15].

## Materials and methods

### Construct engineering

The mkp-1 gene cloned into the pcDNA 3.1 (+) vector with the FLAG tag attached to the C-terminus (tag composition: DYKDDDDK) was purchased at GenScript Biotech Corp. Simultaneously, primers were developed to allow the connection of the double Strep-tag (tag composition: WSHPQFEK) to the construct with the C-FLAG-tag in a multi-step procedure. The primers were purchased at Genomed. The nucleotide sequences coding for the Strep-tagging amino acids were introduced using standard molecular biology techniques: QuikChange site-directed mutagenesis [16], restriction enzyme digestion, *Escherichia coli* cell transformation, isolation of plasmid DNA [17] using commercial kits (Sigma-Aldrich). The correctness of joining the tags has been verified by sequencing the obtained constructs (Genomed).

### Cell culture and generation of cell lines stably expressing MKP-1 with SF tag

PC12 cells purchased from the American Type Culture Collection, were cultured in medium Ham’s F-12K (Thermo Scientific, 21127022), 15% horse serum (Thermo Scientific, 26050088), 5% foetal bovine serum (Sigma-Aldrich, F9665), 1% Pen-Strep (Sigma-Aldrich, P4333) at 37 °C in humidity-saturated 5% CO_2_ atmosphere [18]. Once the cells have reached the appropriate confluence, they were transfected with reagent Escort III (Sigma-Aldrich, L3037), according to the manufacturer’s protocol, to introduce a construct that allows the production of MKP-1 protein with an attached SF tag. G418 (Geneticin) antibiotic (Sigma-Aldrich, A1720) with a final concentration of 500 μg/ml was used as the selection agent. After 4 weeks of PC12 cell culture, several clones were selected in the presence of G418.

### Western blot

The Western blot was carried out by the standard protocol (www.scbt.com/scbt/resources/protocols). Protein concentrations were determined using the Bradford assay [19]. The following four primary antibodies were used: mouse anti-FLAG M2, Clone M2 (Sigma Aldrich, F1804), mouse anti-DUT (Santa Cruz Biotechnology, sc-166856), mouse anti-fascin (Santa Cruz Biotechnology, sc-46675) and mouse anti-drebrin (Santa Cruz Biotechnology, sc-374269). The secondary antibody was mouse IgGκ light chain binding protein (m-IgGκ BP) conjugated to horseradish peroxidase (Santa Cruz Biotechnology, sc-516102).

### SF-TAP purification

Stably transfected PC12 cells were cultured to obtain a sufficient number of cells (~ 5 × 10^8^ cells) to ensure efficient purification of protein complexes formed by the MKP-1 protein.

Cells were harvested and washed with PBS, resuspended in cell lysis buffer (20 mM Tris–HCl pH 7.5, 150 mM NaCl, 1.5 mM MgCl_2_, 1 mM DTT, 5% glycerol, 1% (v/v) Nonidet P-40, Complete Mini Protease Inhibitor Cocktail (Roche)). The suspension was vortexed and incubated at 4 °C for 30 min. on the rotator with 360° rotation. After centrifuging for 10 min. (10, 000 × *g*, 4 °C), the clear supernatants were added to StrepTactin Superflow (IBA Lifesciences, 2-1206-002) and incubated at 4 °C for 3 h on the rotator with 360° rotation. After incubation, the samples were centrifuged (1500 × *g*, 2 min, 4 °C). The beads were washed 4 times with wash buffer I (20 mM Tris–HCl pH 7.5, 250 mM NaCl, 1.5 mM MgCl_2_, 1 mM DTT, 5% glycerol, 0.2% (v/v) Nonidet P-40), each incubating the beads for 5 min and centrifuging (1500 × *g*, 2 min, 4 °C). Next, resins were washed with wash buffer II (100 mM Tris–HCl pH 8.0, 150 mM NaCl, 1 mM EDTA) and centrifuged (1500 × *g*, 2 min, 4 °C). The STREP elution buffer (100 mM Tris–HCl pH 8.0, 150 mM NaCl, 1 mM EDTA, 2.5 mM desthiobiotin (IBA Lifesciences, 2-1000-002)) was added to the beads and incubated at 4 °C for 30 min on a rotator with 360° rotation. After this time, the resins were centrifuged (1500 × *g*, 2 min, 4 °C). The supernatant was transferred to anti-FLAG-M2 affinity gel (Sigma-Aldrich, A2220) and incubated at 4 °C overnight on a rotator with 360° rotation. After overnight incubation, the resins were centrifuged (1500 × *g*, 2 min, 4 °C). The beads were then washed three times with wash buffer II, each incubating for 5 min and centrifuging (1500 × *g*, 2 min, 4 °C). The FLAG elution buffer (30 mM Tris–HCl pH 7.4, 150 mM NaCl, 200 µg/ml FLAG peptide (Sigma-Aldrich, F3290)) was added to the beads and incubated at 4 °C for 3 h on the rotator with 360° rotation to elute the bound proteins. After this time, the resins were centrifuged (1500 × *g*, 2 min, 4 °C).

The same procedure for isolating protein complexes was performed for untransfected PC12 cells that served as a negative control. After purification, SDS-PAGE [20] (4% stacking gel, 12% separating gel) of obtained samples was combined with silver staining.

### Mass spectrometry analysis

The samples were prepared using paramagnetic bead technology based on the Single-Pot Solid-Phase-Enhanced Sample Preparation (SP3) [21] GE45152105050250 and GE65152105050250 SpeedBeads^™^ mixed in a ratio of 1:1 were used. The proteins were reduced with dithiothreitol, alkylated with iodoacetamide, and digested with Trypsin/Lys-C Mix (Promega, V5071). Then the samples were prepared using the Filter Aided Sample Preparation (FASP) method based on the protocol [22]. The measurements were made on a spectrometer with an Orbitrap-type analyzer in combination with an LC system using the nanoESI ionization method (Thermo Scientific). Peptides were desalted and concentrated on the trap column (AcclaimPep-Map100 C18, Thermo Scientific; ID 75 µm, length 20 mm, particle size 3 µm, pore size 100 Å), then they were separated on the analytical column (AcclaimPepMapRLSC C18, Thermo Scientific; ID 75 μm, length 500 mm, particle size 2 μm, pore size 100 Å) at 50 °C in a 90 min gradient of acetonitrile (2%–40%) in the presence of 0.05% formic acid at a flow rate of 200 nl/min or 250 nl/min. The eluting peptides were ionized in a Digital PicoView 550 nanospray source (New Objective) and analyzed by the mass spectrometer in a data-dependent mode using the Top8 method with 35 s of dynamic exclusion. The lock mass option was enabled for survey scans to improve mass accuracy (two masses were specified). The MS and MS/MS spectra were acquired with a resolution of 70,000 and 35,000, respectively. The automatic gain control (AGC) target for MS scans was set to 1e6, for MS/MS scans it was 3e6. The maximum ion accumulation times for the full MS and the MS/MS scans were 120 ms and 110 ms, respectively. The QCloud quality control system was used for monitoring the performance of the LC–MS/MS instrumentation during the measurements [23, 24].

The presented results come from three different independent experiments (three biological repetitions), but they were measured only once (one technical repetition). The negative control results are from two different independent experiments (two biological repetitions) and were also measured only once (one technical repetition).

Raw data files (samples and negative controls) have been included as Supplementary materials.

The raw data were processed by the Proteome Discoverer platform (v.1.4, Thermo Scientific) and searched against the SwissProt database with *Rodentia* restriction.

The results obtained were analyzed and protein identification was carried out using the MASCOT package. The list of proteins identified by mass spectrometry was analyzed using the UniProt database (www.uniprot.org).

## Results and discussion

### Protein expression

Sequencing of the constructs confirmed the generation of the MKP-1 protein cloned into the pcDNA 3.1 (+) vector with the C-terminal SF-tag. Due to the presence of a catalytic domain with rhodanase activity near the N-terminus of the MKP-1 protein [8], a construct containing the SF-tag at the C-terminus of the phosphatase domain was used to transfect cells. The tag at the N-terminus, despite its small size, could potentially disrupt the protein–protein interactions under study and lead to false identification results.

A stably transfected PC12 line was derived. It was decided to perform a stable transfection because it allows obtaining a more uniform culture and physiological conditions, which reduced the risk of non-specific interactions in the experiments performed. Transient transfection is associated with high stress for cells and therefore causes high expression of chaperone proteins, which could hinder the analysis of mass spectrometric results.

**Fig. 1:**
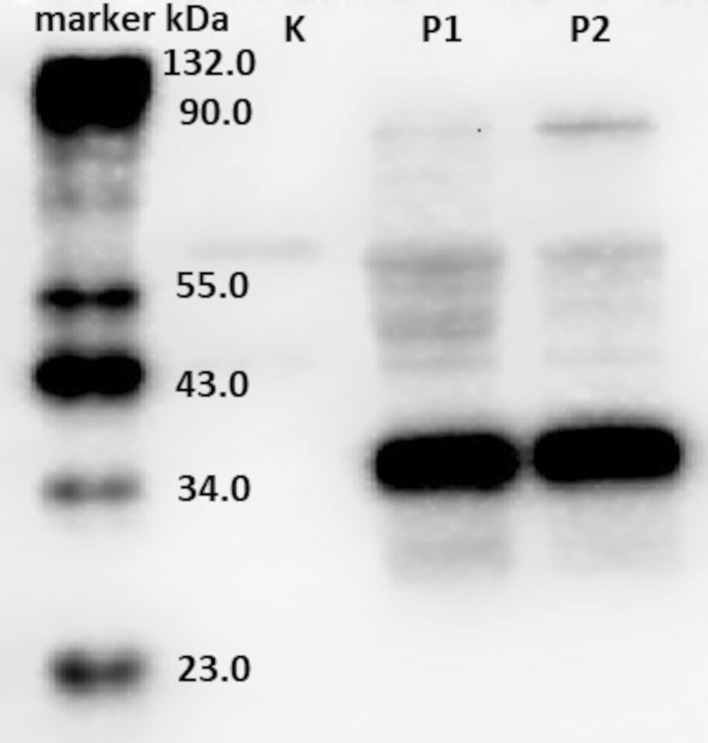
Western blot analysis of the cell lysates. Marker, Cruz Marker^™^ Molecular Weight Standards: sc- 2035 (Santa Cruz Biotechnology); *K* negative control, untransfected cells; *P1* first of the selected clones (transfected cells); *P2* second of the selected clones (transfected cells)

Western blot of selected clones from cell cultures (after transfection and G418 antibiotic selection) using the anti-FLAG antibody gave positive results (molecular weight of MKP-1 is equal to 39 kDa) (Fig. 1).

### Isolation and identification of MKP-1 interacting proteins

Modifications to the TAP method have been successfully introduced into mammalian cell culture (PC12 cells), enabling efficient isolation of specifically interacting proteins. Optimal separation and elution conditions, buffer compositions, incubation times, and separation temperatures were established experimentally to obtain reproducible results and increase the validation of the method.

**Fig. 2:**
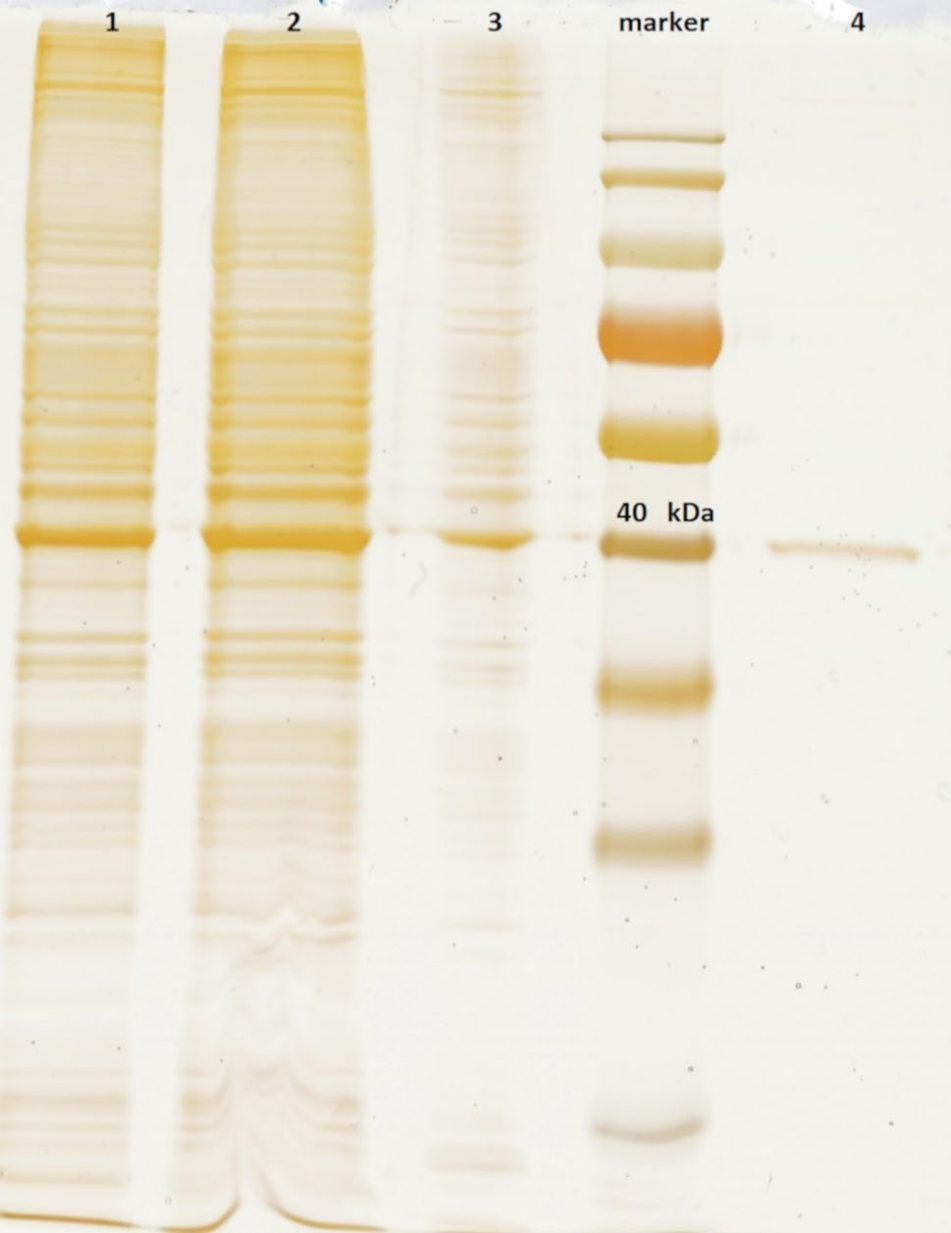
12% SDS-PAGE with silver staining after TAP procedure. Lane 1—total cell lysate, 2—clear total cell lysate (after centrifugation), 3—eluate after STREP purification, Marker PageRuler Prestained Protein Ladder, 26616 (Thermo Scientific), 4—eluate after FLAG purification

After silver staining, a band at around 40 kDa was observed in samples, which corresponds to the size of the MKP-1 protein—39 kDa (Fig. 2). A band for MKP-1 protein is visible in the samples taken after elution from the anti-FLAG M2 agarose, which may indicate the high specificity of the tandem affinity chromatography method.

A comparative analysis of proteins present in positive transfected cultures and negative control was performed. When analyzing the results, only the proteins with the highest amount of peptides were taken into account, on the basis of which the given protein with high percentage coverage of the sequence was identified. The list of proteins identified by mass spectrometry was analyzed using the UniProt database. Based on the available information, the proteins are grouped according to their function. 35 proteins were identified in this way. The results are shown in Table 1.

**Table 1:** Comparative analysis of proteins identified for PC12 cells transfected with the construct MKP-1 with SF tag construct versus negative control (untransfected PC12 cells)

Protein ID	Protein name	Protein function
Q68FP1	Gelsolin	Cytoskeletal proteins
Q9Z1P2	Alpha-actinin-1	Cytoskeletal proteins
Q9QXQ0	Alpha-actinin-4	Cytoskeletal proteins
P85845	Fascin	Cytoskeletal proteins
C0JPT7	Filamin A	Cytoskeletal proteins
Q63610	Tropomyosin alpha-3 chain	Cytoskeletal proteins
P09495	Tropomyosin alpha-4 chain	Cytoskeletal proteins
P16086	Spectrin alpha chain, non-erythrocytic 1	Cytoskeletal proteins
B2GUZ5	F-actin-capping protein subunit alpha-1	Cytoskeletal proteins
Q5XI32	F-actin-capping protein subunit beta	Cytoskeletal proteins
P52944	PDZ and LIM domain protein 1	Cytoskeletal proteins
P85970	Actin-related protein 2/3 complex subunit 2	Cytoskeletal proteins
Q7M0E3	Destrin	Cytoskeletal proteins
Q07266	Drebrin	Cytoskeletal proteins
P45592	Cofilin-1	Cytoskeletal proteins
Q62627	PRKC apoptosis WT1 regulator protein	Cytoskeletal proteins
Q62736	Non-muscle caldesmon	Cytoskeletal proteins
A0A0H2UHM5	Protein disulfide isomerase	Enzymes
P70583	DUT pyrophosphatase	Enzymes
P17751	Triosephosphate isomerase	Enzymes
Q63716	Peroxiredoxin-1	Enzymes
P19945	60S acidic ribosomal protein P0	Ribosomal proteins
P19944	60S acidic ribosomal protein P1	Ribosomal proteins
P02401	60S acidic ribosomal protein P2	Ribosomal proteins
P49242	40S ribosomal protein S3a	Ribosomal proteins
P23358	60S ribosomal protein L12	Ribosomal proteins
P13383	Nucleolin	Nucleic-acid binding protein
Q66HF9	Leucine-rich repeat flightless-interacting protein 1	Nucleic-acid binding protein
P13084	Nucleophosmin	Nucleic-acid binding protein
Q4QR85	Methylosome protein 50	Nucleic-acid binding protein
B2GUV7	Eukaryotic translation initiation factor 5B	Nucleic-acid binding protein
P06761	78 kDa glucose-regulated protein	Chaperones
P82995	Heat shock protein HSP 90-alpha	Chaperones
P04256	Heterogeneous nuclear ribonucleoprotein A1	Others protein
B5DEL1	BTB/POZ domain-containing protein KCTD5	Others protein

Identification of several proteins in the samples: DUT pyrophosphatase, fascin and drebrin were confirmed by Western blot (data not presented).

The largest group among the identified proteins are cytoskeleton proteins. It has been proven that some neuropsychiatric diseases are associated with abnormal structure and atrophy of dendrites that may result from cytoskeleton disorders. Increasing evidence indicates a change in depression associated with the cytoskeleton [25–27].

Chronic stress is one of the important risk factors for depression. It has been shown that during stress, an increase in glucocorticosteroid levels causes dendritic remodelling [28]. Changes in post-translational modifications of tubulin isoforms that can modulate microtubule dynamics have been observed in animal models of depression undergoing CUS (chronic unpredictable stress) [29].

#### Actin and actin-binding proteins

Actin is a highly conserved protein that polymerizes to produce filaments that form cross-linked networks in the cytoplasm of cells. Actin exists in both monomeric (G-actin) and polymeric (F-actin) forms, both forms playing key functions, such as cell motility and contraction. In addition to their role in the cytoplasmic cytoskeleton, G- and F-actin also localize in the nucleus, and regulate gene transcription and motility and repair of damaged DNA. [30]. A variety of actin-binding proteins influence the structure and organization of the actin cytoskeleton [31].

Actin's involvement in neurobiological processes associated with stress and mood disorders is probably due to the role of this protein and its regulators in neuroplasticity, in particular in excitatory synapses, as well as in anchoring postsynaptic receptors in hippocampal neurons [25].

Proteomic studies have shown that actin changes its expression levels in the hippocampus in rats after treatment with various antidepressants, which confirmed the role of microfilaments in molecular mechanisms of depression [32]. Several binding proteins and actin regulators, including cofilin-1, have also been found to be up-regulated in various animal models of depression.

#### Cofilin-1 and drebrin

Cofilin-1 and drebrin are actin regulators involved in the signalling pathway mediating synaptic plasticity. Cofilin 1 binds to F-actin and exhibits pH-sensitive F-actin depolymerizing activity [33].

Drebrin is actin cytoskeleton-organizing protein that plays a role in the formation of cell projections [34]. It has been found to modulate glutamatergic and GABAergic activity in hippocampal neurons. Some studies indicate that disturbances in the actin regulation mechanism (decrease in drebrin level and increase in dephosphorylated cofilin) result in synaptic dysfunction. In addition, drebrin level was found to negatively correlate with the severity of cognitive impairment. Gene expression analysis in rats showed that antidepressants can reduce the level of drebrin expression in the hippocampus [32].

#### Filamin A

Filamin A is an actin-binding protein, which participates in the formation of the cytoskeleton, anchors a variety of proteins in the cytoskeleton and regulates cell adhesion and migration [35].

It is believed that the pathophysiology of depression includes, among other things, the malfunctioning of the D2 dopamine receptor, as well as the reward system functions. It has been found that filamin A is present in the dopamine receptor D2 and D3 expressing neurons and may be involved in their dysfunction [36].

#### Caldesmon

Caldesmon is a calmodulin-binding protein. It is an actin- and myosin-binding protein implicated in the regulation of actomyosin interactions in smooth muscle and nonmuscle cells (could act as a bridge between myosin and actin filaments) [37].

Almost all cytoskeleton proteins are substrates of one or more kinases. Phosphorylation by intracellular kinases may be a common feature of many actin-binding proteins. Through phosphorylation, the binding properties of actin by these proteins change, thus enabling the reconstruction of the cytoskeleton. Caldesmon phosphorylation causes its activation [38]. The enzymes responsible for this modification are kinases belonging to MAPK, mainly ERK [39]. Although the interaction of caldesmon with MKP-1 has not been discovered so far, it is known that this protein is involved in the MAPK kinase signalling pathways. It can be postulated that increased levels of phosphatase indirectly affect caldesmon functions. This effect may be due to the inhibition of ERK kinase by dephosphorylation.

Among other of the identified partners interacting with the MKP-1, there are ribosomal proteins, chaperones, nucleic acid binding proteins and enzymes.

It has been shown that antidepressant treatment can affect some of the chaperone proteins in the hippocampus of animals showing a depressive phenotype [39].

At present, however, it is unknown whether other identified proteins may be involved in the pathophysiology of neuropsychiatric diseases.

## Conclusions

It is worth noting, however, that there is not much information found in the literature regarding the confirmed protein–protein interactions formed by the MKP-1 phosphatase. On the other hand, proteins identified in the present work have been reported in the literature concerning studies on depressive disorders, which speaks in favour of their role in depression. The analysis of the results should also take into account the fact that increased MKP-1 expression, besides its main enzymatic activity, may have an indirect effect on changing the profile of interacting proteins in cells, which in turn may result in a depressed phenotype. Therefore, we suggest that the proteins identified in the present study may potentially be involved in the molecular mechanism of depression.

## Supplementary Information

Below is the link to the electronic supplementary material.

Supplementary file1 (RAW 1392923 kb)

Supplementary file2 (RAW 792503 kb)

Supplementary file3 (RAW 979808 kb)

Supplementary file4 (RAW 926008 kb)

Supplementary file5 (RAW 1389959 kb)

## Data Availability

Raw data files have been included as Supplementary materials.

## Abbreviations

CUS: Chronic unpredictable stress
ERK: Extracellular signal-regulated kinase
FASP: Filter aided sample preparation
G418: Geneticin
GABA: Gamma-aminobutyric acid
MAPK: Mitogen-activated protein kinase
MKP-1: Mitogen-activated protein kinase phosphatase-1
PBS: Phosphate buffered saline
PPIs: Protein–protein interactions
SF-tag: Tag consists of a tandem Strep-tag II and a FLAG epitope
SP3: Single-pot solid-phase-enhanced sample preparation
TAP: Tandem affinity purification
